# Soft Dopants for a Soft Lattice: Toward Electronic Doping Control in Metal Halide Perovskite Quantum Dots

**DOI:** 10.1021/acsmaterialsau.6c00196

**Published:** 2026-10-12

**Authors:** Ole F. Dressler, Sure Ceylan, Nicolas Borter, Maksym V. Kovalenko

**Affiliations:** † Laboratory of Inorganic Chemistry, Department of Chemistry and Applied Biosciences, ETH Zürich, CH-8093 Zürich, Switzerland; ‡ Laboratory for Thin Films and Photovoltaics, Empa—Swiss Federal Laboratories for Materials Science and Technology, CH-8600 Dübendorf, Switzerland

**Keywords:** quantum dots, electronic doping, quantum confinement, optical spectroscopy, metal halide perovskites

## Abstract

Electronic doping control in semiconductors is essential for engineering optoelectronic properties yet remains elusive for metal halide perovskite quantum dots (MHP QDs). This perspective assesses the current state of aliovalent substitutional doping in MHP QDs and concludes that the softness of the MHP lattice drives both native defect compensation and thermodynamic phase separation, rendering conventional inorganic dopants ineffective. Instead, we propose soft organic dications as a viable new class of persistent donor dopants for MHP QDs. The distinct optical signatures of majority carriers in QDs make optical spectroscopy a powerful screening tool for electronic doping. In a case study of natively intrinsic and self-p-doped MHP QDs, the orthogonal optical responses upon post-synthetic A-site substitution provide first indications for organic dications as viable donor dopants.

## Graphical abstract

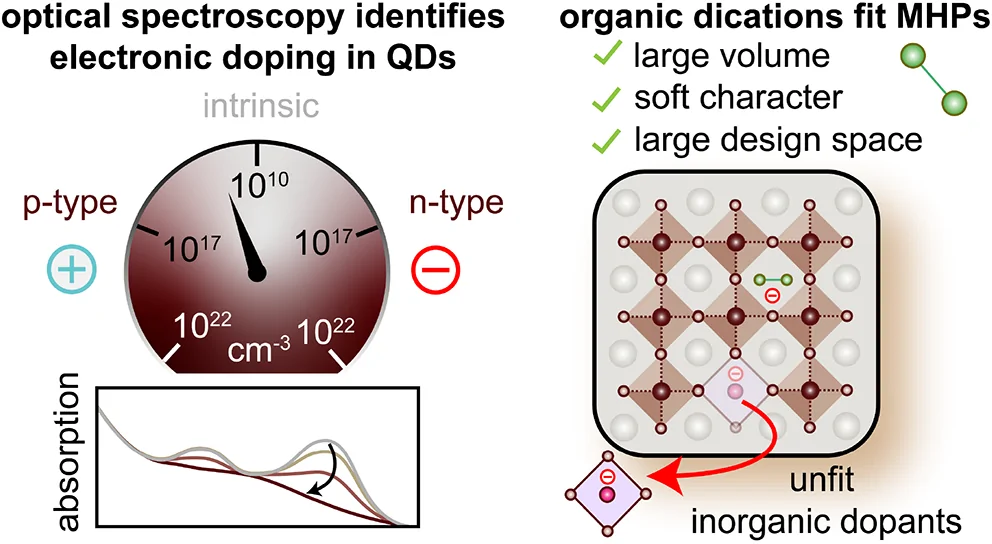

The facile synthesis and solution processability of metal halide perovskite quantum dots (MHP QDs) combined with their narrow emission line widths, high photoluminescence quantum yields without epitaxial shelling, and tunable bandgaps have placed them amongst the most promising materials for next-generation optoelectronic applications.^1,2^ Their potential was demonstrated for LEDs,^3^ lasers,^4^ solar cells,^5^ and quantum communication technologies,^6^ yet the very basis of semiconductor engineering, which is electronic doping, is still not established.^7–9^

Electronic doping in semiconductors is the emergence of a majority carrier population from charged defect states, resulting in a shift of the Fermi level away from the middle of the bandgap.^10–12^ The manipulation of mobile charge-carrier concentrations in semiconductors is essential for controlling their properties, such as (non-)­radiative carrier recombination, photon absorption, and charge injection/extraction at interfaces or junctions.^13,14^ A common strategy for introducing the required charged defect states is aliovalent substitution.^14^ Substitution with higher valence heteroatoms generates donor levels which, upon ionization, provide mobile electrons to the conduction band yielding n-type semiconductors (Figure 1). Conversely, occupying lattice sites with lower valence heteroatoms allows electrons from the valence band to ionize onto these acceptor states giving a majority mobile hole population in p-type semiconductors (Figure 1). Notably, substitution by isovalent atoms cannot alter electronic doping. For intrinsic semiconductors, the Fermi level lies at the center of the bandgap, with equal electron and hole concentrations. Pure silicon, as well as lead-based MHPs, have intrinsic carrier concentrations of ∼10^10^ cm^–3^ at room temperature.^14–17^ Doping can raise the majority carrier concentration by many orders of magnitude, up to 10^17^ cm^–3^ for conventional semiconductor applications, above which materials are considered semimetals.^14^ When the Fermi level is moved into the bands, the material becomes degenerately doped with metallic character and majority-carrier concentrations of 10^22^ cm^–3^ or above.^14^ Notably, tin-based MHPs are natively self-p-doped and undergo further p-doping upon oxidation, such that near-metallic conductivities were reported.^18–20^

**Figure 1:**
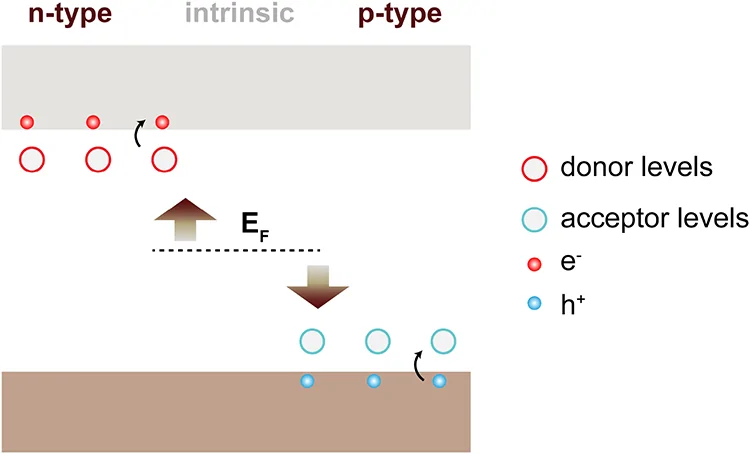
Schematic of electronic doping in semiconductors, which involves the incorporation of aliovalent dopants that can be ionized to produce a majority carrier population and move the Fermi level closer to the band edges.

In the field of MHP QDs, the term “doping” is generally associated with a broad range of phenomena, including the insertion of emissive or magnetic impurity ions or the redox alteration of the material. For example, isovalent substitution of Pb^2+^ with Mn^2+^ gives rise to a broad emission band associated with d-shell intraatomic transitions, fueled by exciton-to-dopant energy transfer,^21^ but is not expected to alter the host’s electronic doping level and has not been reported to do so. While applying a voltage modifies the Fermi level and can lead to the temporary injection of charges into QDs, it does not provide permanent majority-carrier populations.^22–24^ Surface adsorption of molecules with electron-donating or withdrawing groups has moved the Fermi level of bulk perovskites by charge-transfer between the molecule and the semiconductor, but represents a transient surface effect and does not alter the electronic state of the crystal lattice.^25,26^ In the conventional sense of sustained electronic doping by aliovalent atomic substitution, MHP QDs remain essentially undopable.

In this Perspective, we first discuss how optical spectroscopy observes the presence of majority carriers in QDs as bleaching of the discrete excitonic absorption and quenching of the photoluminescence (PL)—signatures that have not been reported to date for MHP QDs. We posit that the A-site is inaccessible to inorganic dications due to size mismatch, whereas B-site substituents form deep traps or are expelled by the natively soft lattice, leaving soft organic cations as the most promising unexplored class of donor dopants. In this Perspective, we highlight the smallest organic dication ethylenediammonium (EN), which was successfully incorporated into efficient hybrid tin-based MHPs solar cells,^27,28^ as potential electronic donor dopant for MHP QDs. In an experimental illustration, the addition of EN to CsPbBr_3_ QDs causes absorption bleaching and PL quenching, resembling the optical signatures of n-doping. Conversely, both the excitonic absorption and PL intensity are enhanced in CsSnI_3_ QDs, consistent with the reduction of its inherent self-p-doping. We finally outline applications of electronic doping control in MHP QDs.

## Optical Spectroscopy of Doped QDs

Identifying promising dopant candidates requires facile methods that effectively probe the changes in electronic doping. X-ray or UV photoelectron spectroscopies for determining the Fermi level are not commonplace in synthetic laboratories. Charge transport measurements by direct methods (conductivity), typically as a field-effect transistor or Hall-effect configuration, require invasive manipulation with a stripping or exchange of the insulating organic ligand shell.^29^ Terahertz spectroscopy yields the frequency-dependent complex conductivity, from which carrier concentrations can only be extracted by modeling carrier mobility.^30^ Fortunately, high-doping-level majority-carrier populations have a distinct effect on the fundamental optical properties of confined QDs.^31^ Furthermore, absorption and PL measurements are readily available in synthetic laboratories. While the obtained spectra do not provide a direct measure of the doping levels, they carry qualitative signatures of doping sufficient for initial screening.

The spatial confinement of QDs accelerates Auger-Meitner recombination, in which the energy is transferred to a third carrier instead of being released as a photon.^32–34^ The majority carriers in doped QDs readily serve as the third carrier, providing an efficient non-radiative decay pathway that quenches the PL signal (Figure 2a,b).^31,35^ However, PL quenching is not exclusive to doping; it can also arise from the emergence of electronic trap states due to degradation or loss of capping ligands.

**Figure 2:**
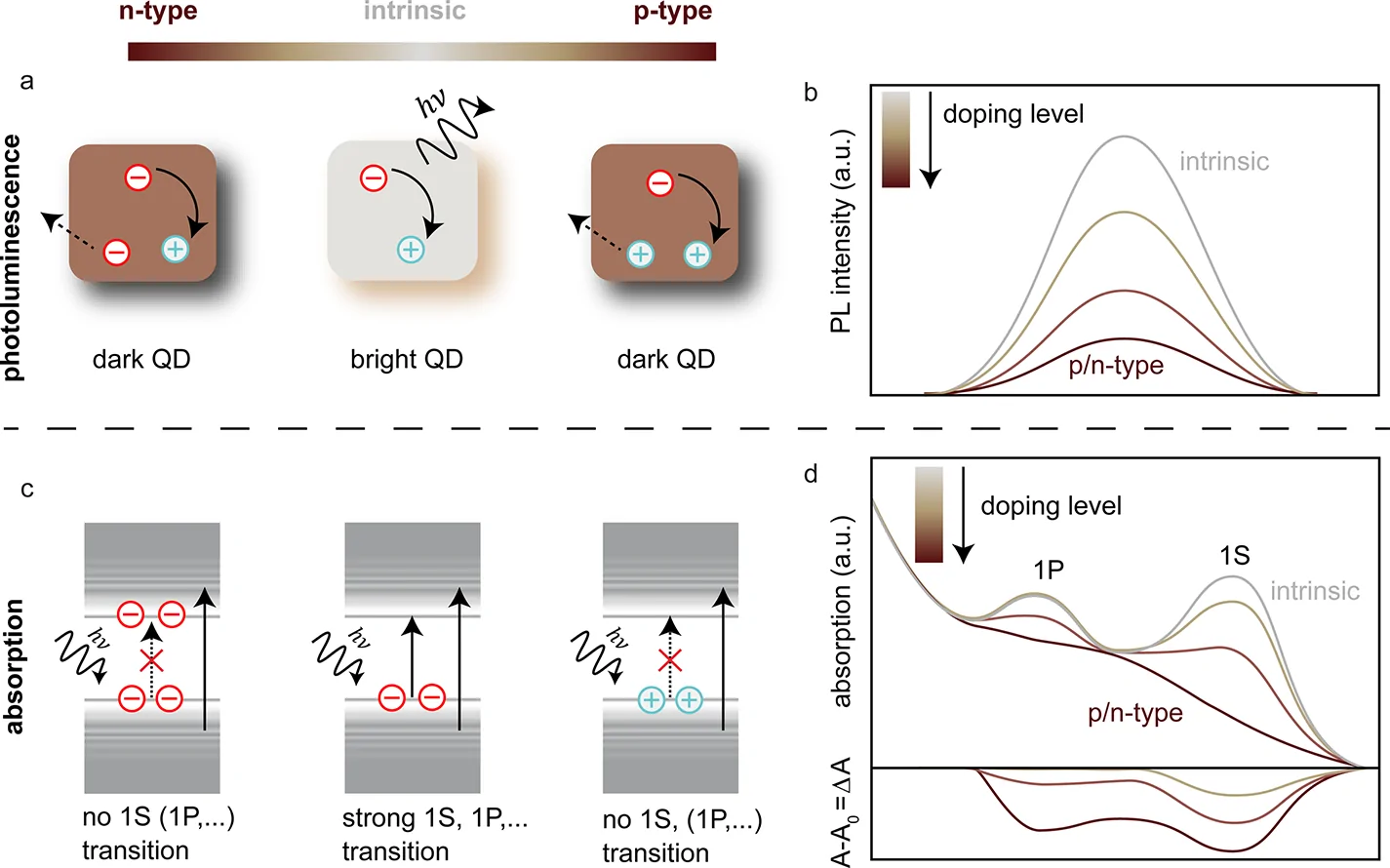
Illustration of the features expected in optical spectroscopy of doped semiconductor QDs: (a) schematic of the radiative recombination of excitons in intrinsic (non-doped) QDs but non-radiative recombination in n/p-type QDs due to Auger-Meitner scattering, (b) schematic of the expected PL curves annotated with their hypothetical doping state showing a decrease in intensity upon electronic doping; (c) schematic of the simplified band energy diagrams of QDs where n/p-type causes empty/full discrete ground/excited states which blocks the lowest-energy transition(s), (d) schematic of the expected absorption curves annotated with their hypothetical doping state showing reduced excitonic absorption upon doping, selective bleaches can be revealed from the differential curve relative to the undoped material.

Quantum confinement causes the band edges of semiconductor QDs to evolve into discrete, atom-like states, with degeneracies governed by the orbital symmetry of the constituent bands.^36–39^ The highest valence band quantum state is denoted 1S_h_ and the lowest conduction band quantum state 1S_e_.^39–41^ The transition between the filled 1S_h_ and empty 1S_e_ gives rise to the 1S excitonic absorption feature characteristic of the nature and size of the QD.^39^ N-doping fills the 1S_e_ and p-doping empties the 1S_h_. Depending on the QD size and state degeneracy, state-filling starts to become significant for carrier concentrations above *ca*. 10^17^ cm^–3^. Subsequently, the Pauli exclusion principle forbids the 1S transition, resulting in a bleach of the lowest excitonic transition of doped QDs (Figure 2c,d).^31,40,41^ For n-type CdSe, two mobile electrons will fill the twofold spin-degenerate 1S_e_, bleaching the first excitonic transition, while six additional electrons are required to fill the threefold orbitally degenerate 1P_e_, bleaching higher transitions.^41^ In the case of MHPs, both the 1S_h_ and 1S_e_ are only spin-degenerate, leading to equivalent Pauli-blocking for two majority electrons or holes per QD.^22,42^

As the excitonic absorption features can also be broadened by size dispersion, the doping-induced bleaches are most cleanly resolved in differential absorption Δ*A* (Figure 2d). In-situ differential absorption is already widely used to evaluate the doping of semiconductor QDs upon electrochemical charge injection,^43^ as recently demonstrated for MHP QDs,^22,23^ but uncommon for evaluating substitutional dopant candidates.

## Particularities of a Soft Lattice

The near-zero formation free energies of MHPs ranging from 0 to −15 kJ mol^–1^ are in stark contrast to <−50 kJ mol^–1^ for conventional semiconductors.^44^ In particular, the enthalpy of formation of the soft MHP lattices is low, favoring easy dissociation and reformation of the material.^44^ Therefore, dopant impurities will be promptly expelled from MHPs by phase-separation unless the substitutional enthalpies are favorable. Conversely, conventional semiconductors with higher lattice enthalpies can be kinetically trapped in a dopant-containing metastable state. Due to the short distance from core to surface, phase-separation is even more pronounced for QDs.^45^ We observed such behavior in unpublished experiments, where in an attempt of doping CsPbBr_3_ QDs with Bi^3+^, the dopant simply phase-separated as Cs_3_Bi_2_Br_9_. Therefore, the thermodynamics of competing dopant phases becomes the limiting factor, defining our criterion for soft dopants in a soft host lattice.

Furthermore, the soft lattice also results in low formation energies of native defects.^7,44^ This poses a fundamental challenge to electronic doping control, as the charge of the aliovalent dopant defect is simply compensated by native defects of the opposite charge (Figure 3) rather than the intended free carriers.^7^ Notably, the native defects in the absence of dopants can also result in n- or p-type semiconductors, and the equilibrium is governed by the defect formation energies. While for most lead-based MHPs defects of both charges are balanced, tin-based contenders suffer from so-called self-doping.^46^ As the formation energies of cation defects are much lower than those of halide vacancies, tin halide perovskites (THPs) are inherently strongly p-type.^19,46^ The resulting near-metallic conductivities^18–20^ have prevented THPs from being more extensively used as lead-free alternatives despite their favorable band gaps in the near-infrared. To that end, electronic doping control through aliovalent substitution promises to cope with the prohibitive self-p-doping.

**Figure 3:**
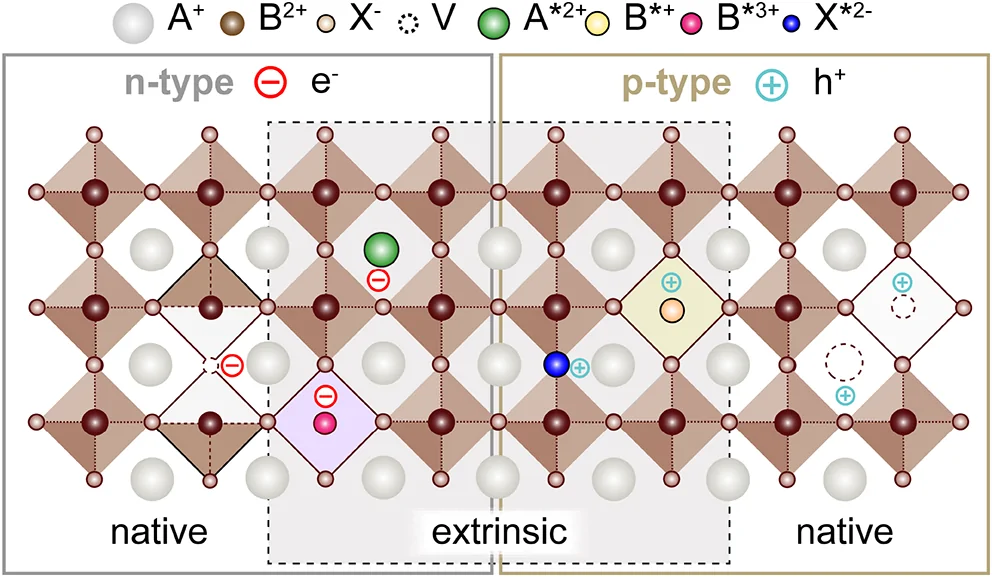
Schematic of the archetypal MHP ABX_3_ structure with various doping scenarios and the corresponding charge-compensating holes and electrons. Vacancies of the perovskite constituents act as donors (anions) and acceptors (cations), which can cause native electronic doping of the material and compete with aliovalent substitution by extrinsic dopants (marked with *) for charge compensation. A*^2+^ and B*^3+^ substitutions form donor states, while B*^+^ and X*^2–^ can act as acceptors.

Furthermore, the electrochemical stability window of MHPs sets limits on the doping levels that can theoretically be achieved. Notably, lead reduction was reported at potentials of −1.0 V vs Ag/AgCl, which lies within the bandgap,^47^ thus prohibiting degenerative n-doping.

## Aliovalent Substitutional Doping Pathways

The ternary ABX_3_ perovskite structure offers distinct doping pathways at each lattice site (Figure 3, Table 1): n-type doping can be achieved through divalent A-site or trivalent B-site donors, while p-type doping requires acceptors formed by monovalent B-site or divalent X-site substitution. The geometric framework of the perovskite structure, governed by the Goldschmidt tolerance factor, restricts suitable substituents based on ionic radii.^48^ For A-site substitution, even the largest inorganic dication, Ba^2+^ (161 pm), falls significantly short of the smallest MHP A-site cation, Cs^+^ (188 pm), a mismatch of ∼15% that pushes the tolerance factor to the boundary of structural stability.^49^

**Table 1:** Kroeger-Vink Notations for the Aliovalent Substitutional Doping Pathways Sketched in Figure 3 Where the Extrinsic Dopants are Denoted with Stars

Lattice site	n-type (donors)	p-type (acceptors)
A	A^*2+^: A_A_^*•^ + *e* ^–^	none
B	B^*3+^: B*_B_^•^ *+ e* ^–^	B^*+^: B*_B_^′^ + *h* ^+^
X	none	X^*2–^: X*_X_^′^ + *h* ^+^

Conversely, the similar ionic radii of many mono-, di- and trivalent metal ions to Pb^2+^ or Sn^2+^ mean size compatibility for both donors and acceptors. To that end, trivalent metal donors such as Bi^3+^ have indeed shown an increase in conductivity of MAPbBr_3_ single crystals that is indicative of n-doping.^50^ However, bismuth also enhances non-radiative recombination.^51^ Furthermore, attempts in QDs have shown no bleached bandgap absorption, or, in our experiments, even phase separated.^52^ Overall, the results on Bi doping of MHPs remain ambiguous and need further investigation. Computational screening suggests the formation of shallow defect states for trivalent lanthanide donors.^53,54^ Experimentally, however, the incorporation of lanthanides into CsSnI_3_ thin films has only shown very small changes in conductivities.^54^ Furthermore, acceptor substitution of the B-site of CsPbBr_3_ QDs with, for example, silver has shown no signs of a bleach of the absorption features.^55^ As no reports exist on substituting halides with chalcogenides, structural and thermodynamic considerations mandate exploring alternative dopant classes.

## Soft Organic Dications as Aliovalent Substituents

MHPs are notable for their design space that extends beyond inorganic ions as the large A-site can readily be filled by organic ions due to their flexibility and dynamic tumbling at room temperature.^17,56,57^ Notably, monovalent organic ions such as formamidinium and methylammonium form stable perovskite structures without phase-separating into the halide salts.^17,56,57^ Ions exceeding the size limits of the Goldschmidt tolerance factor have been reported to form metastable phases as QDs.^58^ The mixing of small monovalent and larger divalent organic ions has even improved the efficiency of tin-based solar cells, forming so-called “hollow” perovskites.^27,28^

To illustrate the optical response of MHP QDs upon addition of an organic dication such as EN, we synthesized CsPbBr_3_ and CsSnI_3_ QDs according to procedures developed in our group,^59,60^ and monitored absorption and PL during the slow addition of dilute EN-oleate. As the two materials have inherently different majority carrier concentrations due to native defects, opposite spectroscopic responses are expected upon identical treatment. The intrinsic CsPbBr_3_ QDs initially exhibit pronounced excitonic absorption features and bright PL. Upon addition of EN, the excitonic absorption is reduced, and the differential absorption spectra show a successive bleach up to the third excitonic transition (Figure 4a). At the same time, the PL is drastically quenched (Figure 4b). Both observations resemble the spectroscopic changes expected for n-doping (Figure 4c). The self-p-doped CsSnI_3_ QDs initially show a barely defined excitonic absorption and low PL intensity. Upon exposure to EN under equivalent experimental conditions as for CsPbBr_3_, the excitonic absorption gets enhanced, visible as enhanced differential absorption (Figure 4d). Furthermore, the PL intensity strongly increases (Figure 4e). These observations align with reports on bulk material where EN has been associated with compensation of the majority hole concentration of the initially p-type material and a shift towards intrinsic character (Figure 4f).^61^

**Figure 4:**
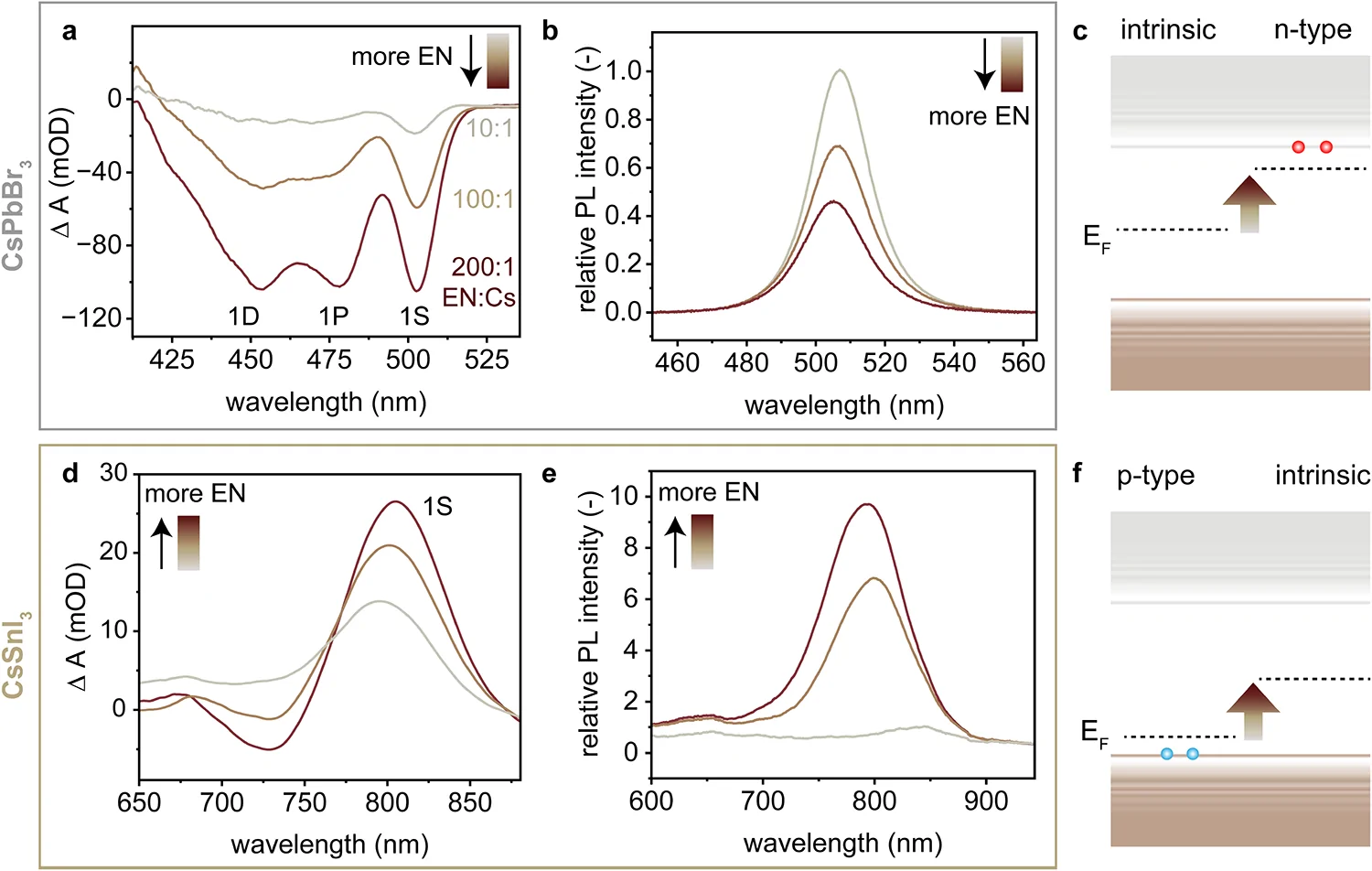
In-situ optical spectroscopy data of MHP QD samples upon the addition of EN-oleate to dilute QD solutions, the EN:Cs ratio is given from the absolute molarity present in the sample. For CsPbBr_3_ QDs the experiment shows (a) a bleach of the first three discrete absorption features in the differential absorption curves given in optical density (OD) along with (b) a quenching of the PL intensity; (c) schematic illustrating the band energy diagram for a material that transitions from near intrinsic to n-type as a postulation for CsPbBr_3_ QDs upon EN addition; for CsSnI_3_ QDs the experiment shows (d) an enhanced absorption near the bandgap together with (e) a strong increase in PL intensity; (f) schematic illustrating the band energy diagram of an initially p-doped material transitioning towards more intrinsic as a postulation for CsSnI_3_ QDs upon EN addition.

Overall, these observations illustrate how optical spectroscopy can distinguish responses for different majority carrier populations, providing an assessment of potential doping strategies. We emphasize the importance of matching the softness of the dopant and host to avoid phase segregation and point to the large design space of organic dications as a promising route to transform electronic doping in this materials class. Beyond EN, cyclic diammoniums such as piperazinium have reportedly reduced dark currents in tin-based solar cells,^62^ and recent experiments have shown the incorporation also of larger linear dications with up to four carbon atom backbones.^63^ However, a limit to the size of the dications can be expected, as larger ions are more likely to induce lattice distortions and promote vacancy formation. Optical spectroscopy can serve as a first indication of doping level changes for these candidates, yet futures studies should also investigate the structural incorporation of the dopant molecules by means of NMR and provide independent evidence of doping through X-ray or UV photoelectron spectroscopy binding energies or the presence or absence of intraband transitions in the infrared.

## Where Electronic Doping Control of MHP QDs is Needed

Controlled majority carrier concentrations are essential for diode-based devices where doped QDs would enable the formation of QD homojunctions. Nonetheless, the insulating ligand shell requires ligand stripping before device integration, which is particularly undesirable for labile MHP QDs. However, electronic doping can directly tune the optical properties of QD active layers, of particular importance given the promise of MHP QDs as quantum and classical light emitters.

Bright spontaneous emission in QDs generally requires low majority carrier concentrations to minimize non-radiative Auger-Meitner losses, and thus a primary goal of electronic doping control in MHP QD systems is to minimize majority carrier populations. For example, controlled electronic doping offers a route towards compensating for unwanted native doping. Overcoming the self-p-doping inherent to narrow-bandgap tin-iodide-based MHP QDs would translate into higher PL quantum yields for this lead-free alternative (Figure 5a) needed for integration into red and near-infrared LEDs, superresolution microscopy, and quantum emitter photonic circuits.

**Figure 5:**
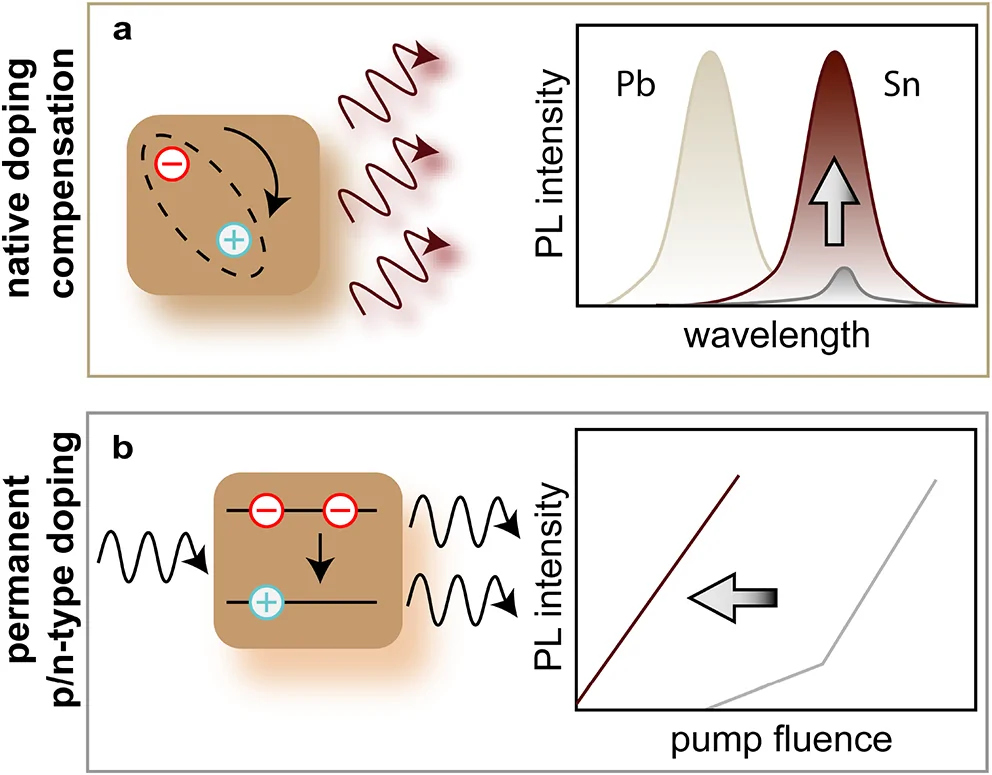
Schematic illustrating the potential applications of electronic doping control in MHP QDs. (a) Bright red and near-infrared THP QDs that are otherwise limited by non-radiative Auger-Meitner recombination due to self-p-doping and (b) ASE in the single exciton regime in doped QDs needed for zero-threshold MHP QD lasers.

Specific optical properties, for example, amplified spontaneous emission (ASE), may benefit from controlled majority carrier concentrations. The discrete nature of the band edges in QDs usually requires pump power to reach the biexciton regime for ASE.^64,65^ However, doped QDs can already achieve population inversion and ASE in the single-exciton regime by circumventing optical transparency (Figure 5b).^64,65^ Fine-tuning the majority-carrier concentration could, in principle, achieve population inversion before optical excitation, producing a zero-threshold laser with improved stability and the potential for continuous-wave operation (Figure 5b).

## Conclusion

Electronic doping control is the cornerstone of semiconductor engineering, yet its realization in MHP QDs has remained elusive. In this perspective, we highlight that in semiconductor QDs, electronic doping manifests in bleaching of the discrete absorption features and loss of PL intensity, which can be used as a powerful tool to screen electronic dopant candidates. MHP QDs are particularly challenging systems for aliovalent substitutional doping due to the low formation energy of compensating native defects and unfavorable inorganic dopant incorporation into the soft lattice. Organic dications, which combine an extensive design space with a large, occupied volume and soft-ion character, are suggested as donor dopant candidates via aliovalent substitution at the A site. Reduced absorption and PL in initially intrinsic CsPbBr_3_ QDs, together with enhanced features in self-p-doped CsSnI_3_ QDs, illustrate optical responses that may arise from donor-induced changes in the carrier population. Electronic doping control could open pathways to bright near-infrared emission from tin-based MHP QDs and zero-threshold lasing.

### Chemicals

Cesium carbonate (Acros, 99%); diisooctylphosphinic acid (fluorochem, 90%); ethylenediamine (Sigma-Aldrich, 99%); hexane (Sigma-Aldrich, >97%); lead­(II) bromide (prepared in house); octane (Sigma-Aldrich, 97%); oleic acid (Ambeed, 99.9%); tin­(II) iodide (Strem, 99%, sublimed); trioctylphosphine oxide (TOPO) (Strem, 99%)

### Synthetic Procedures

The CsPbBr_3_ QDs were synthesized according to literature procedures.^59^ 100 μL hexane and 200 μL PbBr_2_-TOPO 0.04 M (1:5 Pb:TOPO) were loaded into a vial to which 100 μL of CsDOPA 0.02 M were added under strong stirring. After 30s, the reaction was quenched by the addition of 1 mg of branched phosphoethanolamine^66^ dissolved in mesitylene (0.1 g/mL).

The CsSnI_3_ QDs were synthesized according to literature procedures^60^ under inert conditions with dry solvents: 1 mL of hexane and 300 μL SnI_2_-TOPO 0.04 M (1:1 Sn:TOPO) were loaded into a vial, to which 100 μL of Cs-OA 0.02 M were added under strong stirring. After 10s, the reaction was quenched by the addition of 1 mg of branched phosphopropanolamine^66^ dissolved in mesitylene (0.1 g/mL).

The EN solution was prepared from 70 mg ethylenediammonium oleate (prepared in-house from ethylenediamine and oleic acid), 3 mL hexane, and 500 μL additional oleic acid to give a 0.03 M solution.

The EN solution was added via a syringe pump at 0.05 mL/min into a cuvette with a 10–100× diluted QD solution under stirring in a 3D printed PL/Abs setup. The absorption and PL were recorded simultaneously at different heights of the cuvette. The PL spectra were recorded with a compact spectrometer (Thorlabs, CCS200M) with excitation of a 365 nm UV-LED in reflection geometry. The absorption spectra were recorded with a compact spectrometer (Ocean Insight, HDX-XR) and excited by a Deuterium-Halogen lamp (Ocean Optics, DH-2000-BAL).

The absorbance and relative PL intensity were corrected for the dilution by the added volume of EN solution before subtraction from the initial spectrum. The ratio of EN:Cs corresponds to the ratio of the quantity of ENOA_2_ added to the quantity of Cs in the CsPbBr_3_ QDs as determined through their absorbance.^67,68^
